# Control Efficacy of Annual Community-Wide Treatment against *Schistosoma japonicum* in China: A Meta-Analysis

**DOI:** 10.1371/journal.pone.0078509

**Published:** 2013-11-04

**Authors:** Jing Su, Da-Bing Lu, Xia Zhou, Su-Rong Wang, Hong-Xiang Zhuge

**Affiliations:** 1 Department of Epidemiology and Statistics, School of Public Health, Soochow University, Suzhou, China; 2 Department of Parasitology, School of Biology and Basic Medical Sciences, Soochow University, Suzhou, China; Queensland Institute of Medical Research, Australia

## Abstract

**Backgrounds:**

Human schistosomiasis is caused by *schistosome*, with annual loss of over 70 million disability adjusted life years in the world. China is endemic with *Schistosoma japonicum* and large-scale chemotherapy with praziquantel has become the mainstay of control in China since 1990s. However, the control effects of mass treatment in the field have been uneven. Moreover, mass treatment has come into a wide use in other countries with limited health resources. Therefore, a better understanding of the control effect of mass treatment is in an urgent need.

**Methods:**

We performed a systematic search of the literature to investigate the control efficiency of annual community-wide treatment (ACWT, treatment to an entire community without any preliminary screening) with a single dose of PZQ (40 mg kg^−1^ bodyweight) against schistosome in humans in China. Three Chinese literature databases, including China National Knowledge Infrastructure, WanFang and Chinese Scientific Journal Databases, and the PubMed were searched. Pooled prevalence ratios (prevalence after to before treatment) were used to assess effect. Our protocol is available on PROSPERO (No. CRD42013003628).

**Results:**

22 articles were included. Meta-analyses on data from 18 studies on one round of ACWT, 17 studies on two consecutive rounds and 6 studies on three consecutive rounds were performed. The results showed control effects of ACWT plus other measures were statistically significant, with prevalence ratios being 0.38 (0.31, 0.46) for one round, 0.28 (0.22, 0.35) for two rounds and 0.22 (0.10, 0.46) for three rounds. When ACWT was performed alone or with health education only, the values for one and two rounds were 0.389 (0.307, 0.492) and 0.348 (0.300, 0.403), respectively.

**Conclusions:**

The control effect of ACWT alone or with other measures is significant and increases with the number of rounds. Such program is recommended in high endemic areas and the criteria yet merit further assessment.

## Introduction

Human schistosomiasis is caused by *schistosome* including *Schistosoma haematobium*, *S. intercalatum*, *S. mansoni*, *S. japonicum* and *S. mekongi*, a parasite that is the second most important after *Plasmodium* to the public health in tropical and subtropical regions, resulting in 207 million people infections [1] and annual loss of over 70 million disability adjusted life years (DALYs) [2]. A series of control measures, as a consequence, has been developed to reduce schistosome infections and infection-associated morbidity in endemic populations during the last several decades [3], [4], [5], which include health education, snail control, environment management and modification, sanitation and water supply, and large-scale chemotherapy. Due to limited resources or support, unfortunately, not all the approaches have been successfully and widely carried out in endemic areas, particularly in most African countries. Therefore, there seems to be an urgent need in establishment of an effective control program to prevent schistosomiasis in such high endemic areas [6].

In China, schistosomiasis, caused by *Schistosoma japonicum*, has existed for over 2000 years, and it is estimated in the 1950s near 12 million people were infected [7]. After the founding of the new government, a high priority has been given to schistosomiasis control in the public health work and the national control program, aiming to eliminate the disease, was developed and implemented mainly through snail control. Since 1990s with an efficient and high safety treatment available for infected individuals with the drug Praziquantel (PZQ), particularly with a special support from the World Bank Loan Project during 1992–2001 [8], China had then adopted morbidity control as its main aim in most endemic areas, which mainly relied on large scale chemotherapy. After the implementation of such program China has seen a great success in schistosomiasis control in a majority of endemic areas [9]. In 2004, under the context of a rapid development of economy, China set new aims for the disease control and new strategy was formed. The ongoing control program, through integrated measures, has shown promise [10]. More comments or reviews are seen in [11], [12], [13], [14].

It is noted that in China, one of the most important approaches, contributing to the great progress in disease control particularly in areas at the high level of transmission, is pertaining to large-scale chemotherapy with praziquantel. The merits of broad-spectrum schistosomicide, easy and safe application and at a low price for Praziquantel enable the drug the choice of the treatment of targeted populations, such as school-aged children, adults at risk of infection (e.g. fishermen, farmers, irrigation workers, or women in their domestic tasks), or entire communities, across endemic areas in both morbidity and transmission control in China [15]. Indeed, in other countries such as in Brazil [16] and Uganda [17] where large-scale chemotherapy programs with the drug have induced a marked reduction of 40% to 71% in *S. mansoni* infection prevalence.

However, the control effects of large-scale chemotherapy with PZQ in the field have been uneven in China. For instance after one round of community-wide treatment (i.e. treatment of the entire community, regardless of infection or not for single individuals), the prevalence reduction ranged from 21.5% [18] to 87.3% [19]. Moreover, chemotherapy-based mass treatment has, as strongly recommended by the World Health Organization (WHO) [3], [20], come into a wide use in sub-Saharan Africa where *S. haematobium* and *S. mansoni* remain heavy endemic [17], [21], [22], [23]. All these require a better understanding of the accurate control effect of mass treatment with PZQ against schistosome infections. We therefore conducted a meta-analysis of all available literature (in Chinese or English) focused on annual community-wide treatment (ACWT) in China, as this intervention is easily defined in practice and has been performed in the country for decades. The main aim was to provide a comprehensive estimate of the control effect of ACWT in human populations. In addition, an attempt, if data available, to characterize any factors associated with the low preventive efficacy was also made. Such results will be useful for ongoing programs with ACWT or other forms of mass treatment (for example selective population treatment) in endemic areas, and also improve our ability to predict the long-term effect of the approach.

## Methods

### Search strategy and selection criteria

A systematic search of the Chinese literature, including documents published from January 1, 1980 to November 1, 2012, was conducted to gather data on the control efficiency of annual community-wide treatment against schistosomes in field studies in China. Three major Chinese literature databases, including China National Knowledge Infrastructure (CNKI), WanFang Database and Chinese Scientific Journal Database (VIP), were searched for data pertaining to the effect of the mass chemotherapy in the field in China. We did not include Chinese Biomedicine literature database as it has now a link to and covered by VIP database. We applied the following terms (the corresponding Chinese keywords in Pinyin (phoneticism) were given between brackets): “schistosomiasis” or “schistosome” (xuexichong), in combination with “community-wide treatment” (quntihualiao, jitihualiao, daguimohualiao, or kuodahualiao). To retrieve all community-associated research, we here used the four corresponding keywords in Chinese each as a search term. In order to avoid publication bias due to language, PubMed was also searched for literature in English with the term “schisto* and China and (community-based or community-wide or large-scale or mass) and (chemotherapy or treatment or drug administration)”.

The abstracts of each screened publication were read carefully. The references of retrieved studies were searched for articles that were not identified in database searches. All available field interventional studies on the effect of annual community-wide drug administration of PZQ (alone or combined with any other measures) on *S. japonicum* infection in humans were inspected for inclusion. The criteria were set *a priori* to incorporate studies in which: (1) an annual treatment with a single standard dose of PZQ (40 mg kg^−1^ bodyweight) was applied to the entire community; (2) the infection was identified using a stool examination for the whole community or its representative sub-samples, and recorded at baseline (pre-treatment) and after treatment. A single article may contain more than one study, and studies with duplicate publication, extended analysis of previously published studies or no original data were excluded. Studies each were assessed by both reviewers (JS, SW) independently and data were then extracted twice. Any discrepancies were referred to a panel (DL, HZh) for resolution. This systematic review was developed according to the PRISMA guidelines (see Checklist S1) [24], with a protocol previously registered in PROSPERO [25] which is available on http://www.crd.york.ac.uk/PROSPERO/display_record.asp?ID=CRD42013003628.

### Data extraction

We abstracted relevant parasitological and demographic information from each eligible study using a purpose-built Microsoft Excel sheet. Data were read and input directly from text or tables. The extracted data included study information (authors, title of article, year of publication, year of study commencement), ecological setting where the work was implemented, sample size, diagnostic method, coverage of chemotherapy, previous and concomitant control measures.

### Data Analysis

We calculated prevalence ratio (PR)-the ratio of infection prevalence after one or more years (i.e. rounds) of ACWT to infection prevalence before [11]. PR value thus reflects the effect of the program. PR and its 95%CI were listed by study. We calculated pooled estimates with a fixed or random effects model depending on the heterogeneous tests [26]. We measured heterogeneity in effects between studies with τ^2^ (an estimate of between-study variance), Cochran’s Q (a statistic based on the chi-squared test) and H (the square root of the chi-squared heterogeneity statistic divided by its degrees of freedom), and the I^2^ statistics, the last of which describes the percentage of variation across studies that is due to heterogeneity rather than by chance. Generally, if I^2^ is more than 50%, it may indicate an existence of substantial variation between studies and thus a random effects model should be employed. A detailed description of all these measures for application and their relationships can be seen in the work [27].

We explored the potential sources of heterogeneity (and the confounding factors) in the estimates by using univariable and multivariable meta-regression analyses. Additional analysis was also performed for specific population subgroups based on endemic regions and concomitant control measures. The sensitivity analyses were performed by omitting one study at a time and then calculating the combined PR for the remaining studies. Publication bias was statistically examined with Egger regression test [28], [29], and if significant, a Begg test was then conducted. All statistical analyses were done in Stata/SE (version 11.2).

## Results

### Literature searched

**Figure 1:**
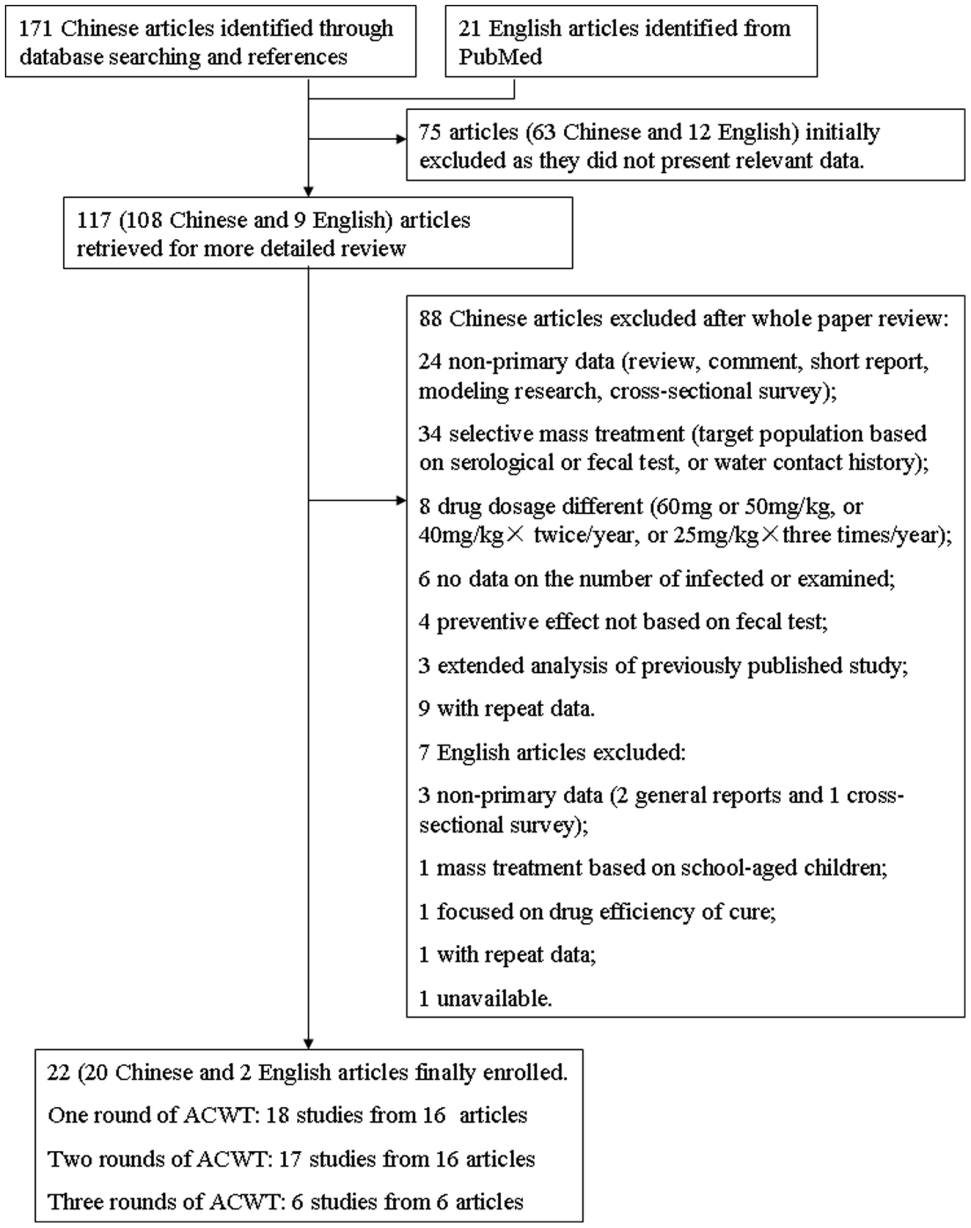
The flow diagram of paper review process. ACWT refers to annual community-wide treatment. One article may contain one or more studies.

Our searches returned a total of 171 records in Chinese, with 169 through database searching and 2 from references. Initial screening excluded 63 articles. After reviewing 108 papers in full, a total of 88 records were ineligible according to criteria and then excluded, which include 24 without primary data (e.g. review, comment or modeling), 34 on selective mass treatment, 8 with drug dosage different than recommended, 6 without data on number of infected and/or examined, 4 with preventive effect not based on fecal test, and 12 with complete or partial repeat data. Finally, a total of 20 documents in Chinese, with potential original data on control effects in field studies in China, were identified. A total of 21 documents in English were retrieved, from which 12 were excluded after initial abstract reading. With the exception of one paper [30], which is unavailable in full text, eight papers were reviewed in full. Three research met the including criteria, but one [31] of these is duplicated with one included Chinese publication [32] and then excluded. The flow diagram in Figure 1 shows the review process, including the number of papers identified and number of documents excluded. A total of 18, 17 and 6 studies from 16 [18], [19], [32], [33], [34], [35], [36], [37], [38], [39], [40], [41], [42], [43], [44], [45], 16 [18], [32], [34], [36], [37], [38], [39], [40], [41], [42], [43], [46], [47], [48], [49], [50] and 6 papers [18], [37], [39], [41], [42], [51] pertaining to control effects of one round, two and three consecutive rounds of ACWT in the field were included, respectively. Out of 24 field research in 22 articles, the concomitant measures reported are generally as follows: 14 with no measures, 1 with health education only, 5 with bovine chemotherapy, and 4 with both bovine chemotherapy and snail control. The details of the included studies are shown in Table S1.

### Meta analysis

**Figure 2:**
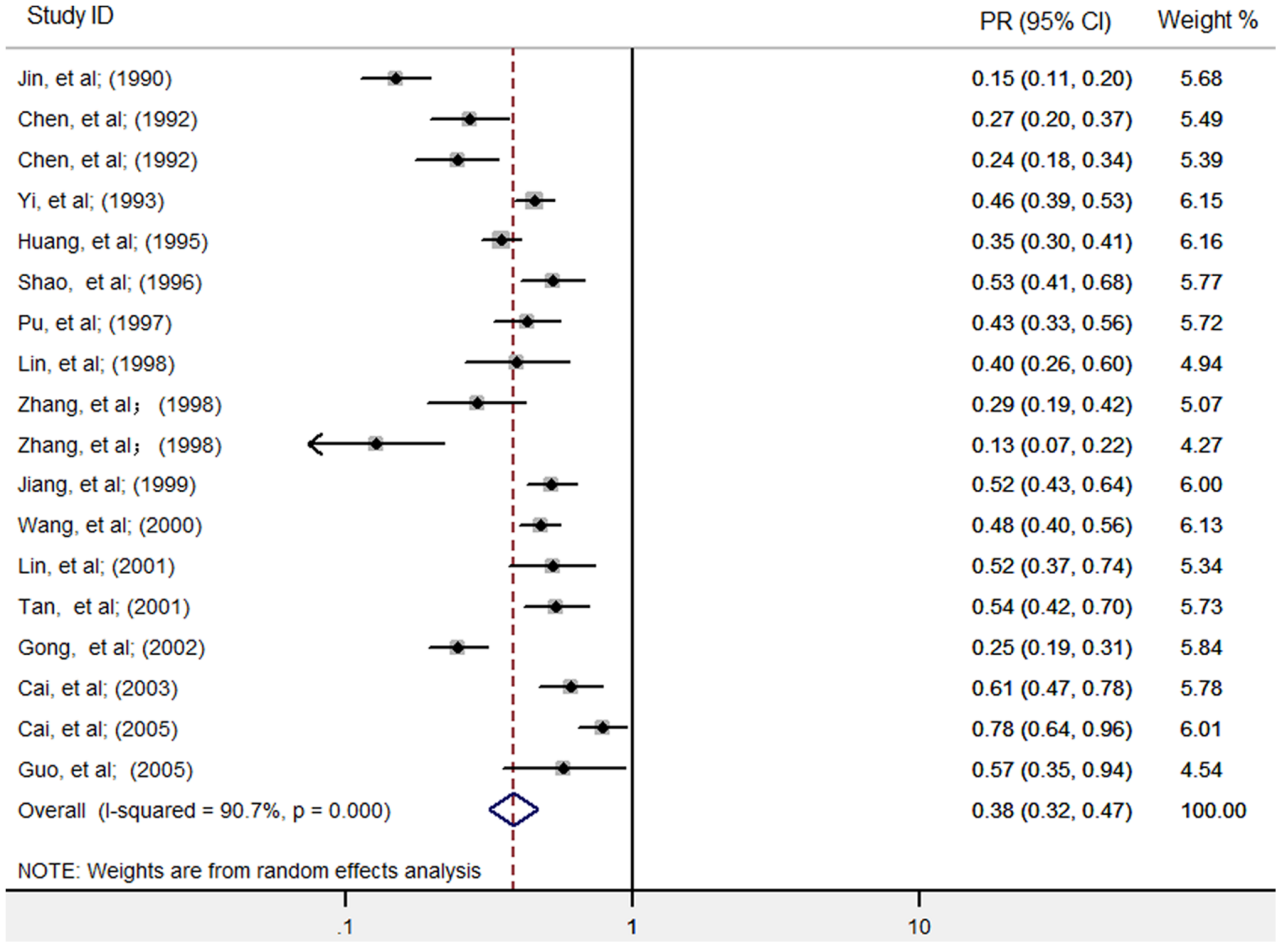
Individual and pooled preventive effects (PRs) of one round of ACWT against schistosome in humans. PR, ratio of infection prevalence after one round of ACWT to prevalence before. ACWT refers to annual community-wide treatment. Diamond and vertical dashed line indicate combined PR, and horizontal lines indicate 95% confidence intervals.

**Figure 3:**
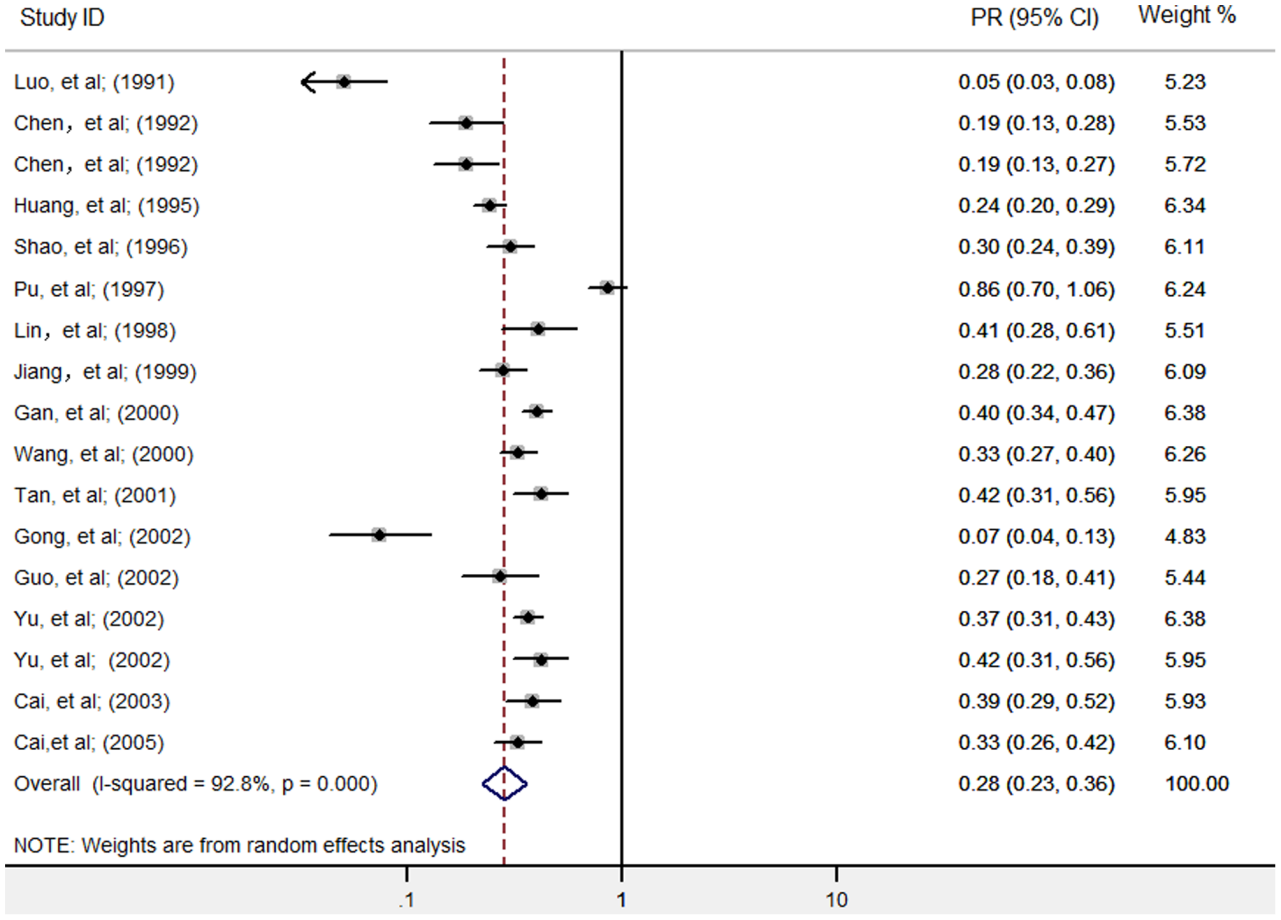
Individual and pooled preventive effects (PRs) of two consecutive rounds of ACWT against schistosome in humans. PR, ratio of infection prevalence after two rounds of ACWT to prevalence before. ACWT refers to annual community-wide treatment. Diamond and vertical dashed line indicate combined PR, and horizontal lines indicate 95% confidence intervals.

**Figure 4:**
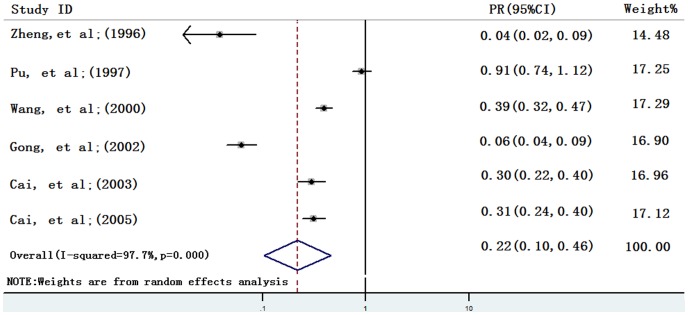
Individual and pooled preventive effects (PRs) of three consecutive rounds of ACWT against schistosome in humans. PR, ratio of infection prevalence after three rounds of ACWT to prevalence before. ACWT refers to annual community-wide treatment. Diamond and vertical dashed line indicate combined PR, and horizontal lines indicate 95% confidence intervals.

As the heterogeneity test indicated substantial heterogeneity between studies within groups of one, two or three consecutive rounds of ACWT (see Table 1), the pooled preventive effects each were analyzed in a random effects model. PR was 0.38 [95% CI: 0.32, 0.47] for one round of ACWT, 0.28 [95% CI: 0.23, 0.36] for two rounds and 0.22 [95% CI: 0.10, 0.46] for three rounds (see Figure 2–4). These show an increasing control effect with the number of rounds of ACWT implemented.

**Table 1:** Heterogeneous test of effects of studies against schistosome infection within each group.

ACWT*	Tau^2^	H (95% CI)	I^2^ (95% CI)	Q	df	P-value
One round	0.15	3.3 (2.8, 3.9)	91% (87%, 93%)	181.16	17	<0.0001
Two rounds	0.21	3.7 (3.2, 4.4)	93% (90%, 95%)	222.54	16	<0.0001
Three rounds	0.82	6.6 (5.4, 8.1)	98% (97%, 98%)	218.12	5	<0.0001

Note: *ACWT refers to annual community-wide treatment.

Sources of heterogeneity between studies on control effects were investigated for one or two rounds of studies with meta-regression. As revealed in Table 2, from individual variable meta-regression analyses, the significantly important factors appeared to be year of publication, coverage and types of endemic regions for one round of ACWT, and the initial infection prevalence, categories of endemic regions (i.e. lake, marshlands and mountains) and concomitant measures for two rounds. The concomitant measures here were classified into three groups: (1) no or health education only, (2) bovine chemotherapy, (3) bovine chemotherapy plus snail control. From a further multivariate meta-regression (i.e. controlling other factors), however, all these relationships each did not remain significant (all P>0.05).

**Table 2:** Univariable analyses of heterogeneity sources of studies with meta-regression.

Sources^¶^	One round of ACWT^*^			Two rounds of ACWT^*^		
	P	Reg. coef.	95% CI	P	Reg. coef.	95% CI
Year	**0.007**	0.0652	0.0204, 0.1099	0.099	0.0642	–0.0136, 0.1420
Porpulation size	0.594	0.0001	–0.0003, 0.0005	0.290	0.0002	–0.0002, 0.0007
Initial prevalence	0.338	–0.9739	–3.0626, 1.1148	**0.001**	–2.8978	–4.4680, –1.3275
Coverage	0.019	3.0758	0.5893, 5.5623	0.820	0.3041	–2.5682, 3.1764
Region						
Lake/Mountains	**0.026**	0.6653	0.0912, 1.2394	**0.012**	0.9719	0.2505, 1.6933
Marshland/Mountains	0.069	0.4678	–0.0416, 0.9772	**0.005**	0.9513	0.3394, 1.5632
Concomitant measures**^§^**						
Measure 1/Measure 3	0.556	0.1928	–0.4892, 0.8747	**0.008**	0.9862	0.3083, 1.6641
Measure 2/Measure 3	0.432	0.3267	–0.5347, 1.1880	**0.018**	0.9954	0.1992, 1.7915

Note: **^¶^** All P>0.05 from multivariate meta-regression.^*^ ACWT refers to annual community-wide treatment. **^§^** Measure 1 refers to no or health education, Measure 2 to bovine chemotherapy, and Measure 3 to bovine chemotherapy plus snail control.

### Subgroup and sensitivity analyses

Subgroup analyses were performed for one and two rounds of ACWT based on categories of endemic regions and main concomitant control measures. As seen in Table 3, the PR values among three categories of endemic regions ranged from 0.260 to 0.516 for one round and 0.138 to 0.371 for two rounds, with a significant difference observed between mountains and lake regions. These values among different concomitant measures varied between 0.320 and 0.440 for one round and between 0.126 and 0.348 for two rounds. The control effect was substantially but not significantly higher for ACWT combined with bovine chemotherapy plus snail control than others. The PR values and 95%CI of ACWT (alone or with health education only) were 0.389 [0.307, 0.492] for one round and 0.348 [0.300, 0.403] for two rounds. More control effects were generally observed for two rounds than for one round.

**Table 3:** Subgroup analyses of prevalence ratio (PR) accroding to endemic regions and concomitant measures.

Group	One round of ACWT^*^					Two rounds of ACWT^*^				
	No. of studies	PR(95%CI)	I^2^	Q	P	No. of studies	PR(95%CI)	I^2^	Q	P
Endemic region										
Lake	5	0.516(0.453, 0.589) **^§^**	0	1.89	0.755	4	0.359(0.309, 0.416) **^§^**	53.10%	6.39	0.094
Marshlands	8	0.419(0.320, 0.548)	90.7%	73.59	<0.001	8	0.368(0.279, 0.486)	92.50%	93.14	<0.001
Mountains	5	0.260(0.168, 0.403)	93.4%	60.25	<0.001	5	0.138(0.070, 0.270)	94.40%	70.91	<0.001
Main concomitant measures										
No or health education	12	0.389(0.307, 0.492)	89.6%	105.98	<0.001	9	0.348(0.300, 0.403)	72.40%	28.95	0.034
Bovine chemotherapy	3	0.440(0.228, 0.849)	94.7%	37.96	<0.001	4	0.351(0.172, 0.715)	95.90%	72.65	<0.001
Bovine chemotherapy and snail control	3	0.320(0.201, 0.510)	91.7%	24.14	<0.001	4	0.126(0.051, 0.311)	95.80%	70.61	<0.001

Note: ^*^ ACWT refers to annual community-wide treatment. **^§^** refers to a fixed effects model, otherwise a random effects model.

The sensitivity analyses showed the combined prevalence ratio, after omitting one study at a time, was not substantially affected by any single study. Note that the summary PRs were all statistically significant (i.e. the upper limit <1) and similar among each other, with a narrow range from 0.368 [95% CI: 0.306, 0.443] to 0.409 [95% CI: 0.345, 0.486] for one round of ACWT, 0.267 [0.219, 0.326] to 0.315 [0.257, 0.385] for two rounds, and 0.163 [0.079, 0.335] to 0.292 [0.161, 0.531] for three rounds. See Figure S1–S3.

### Publication bias

**Figure 5:**
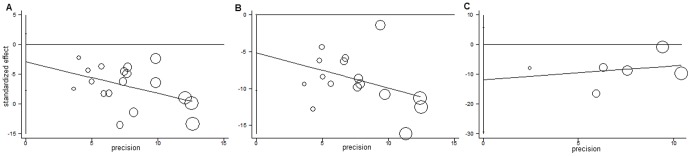
Egger’s publication bias plot. A, B, and C refer to one, two and three rounds of annual community-wide treatment, respectively. The size of circles indicates the sample size of a study.

The linear regression figures, as seen in Figure 5, showed all selected publications normally scattered around the regression line. Egger’s regression test also indicated no (intercept –2.97, P  =  0.207, for one round of ACWT; and intercept –11.93, P  =  0.132, for three rounds) or marginally significant publication bias (intercept –5.13, P  =  0.050, for two rounds). For the last, a further Begg test was performed, which resulted in P  =  0.048.

## Discussion

The present study, to our knowledge, represents the first meta-analysis on control effects of annual community-wide treatment (alone or combined with other measures) against *S. japonicum* in China. No substantial publication bias was detected, suggesting that this meta-analysis was well appropriate in including studies. Coupled by the consistency from sensitivity analyses, our results appeared more reliable. The existence of heterogeneity within each study group suggested that the application of the random effect model was suitable in our meta-analysis.

The approach of large-scale chemotherapy with PZQ has been applied in the field for more than 20 years in China, and it was quite acceptable for the local use in schistosomiasis control owing to its high cure rate and safety and low price [15]. Selective mass treatment, which target populations determined by fecal or serological test, or water contact history through questionnaire, has been one of the most widely used large-scale chemotherapy programs [15], although the sensitivity for identification of true infected individuals for treatment varies among different screening approaches [11]. Here, we focused on the program of annual community-wide chemotherapy (i.e. treatment to the entire community without any preliminary screening) with a single dose, as this definition is quite simple and the extent to which it is carried out could be measured with coverage rate. We retrieved only 22 articles and all were published between 1990 and 2005, mainly due to control strategy performed in China, in which the community-wide chemotherapy program should be modified according to local endemic settings after two to three years or rounds of implementation [15] or replaced with selective mass treatment when infection prevalence is reduced to less than 15% in humans [52].

Our meta-analysis of the included studies demonstrated that the values of prevalence ratios for one to three rounds of annual community-wide treatment (combined with other measures) were 0.38, 0.28 and 0.22, suggesting that the control effect increases with rounds (or years) performed. However, the numbers of studies utilized in our meta-analysis for one or two rounds was almost three times the number of three rounds, which indicated the results from the former are more stable and reliable. It is not a surprise that few studies available on three rounds of mass chemotherapy. Besides the above mentioned treatment control policy change, the compliance rates in humans may decrease over time [45], which, plus other factors or concerns from community residents [53], [54], makes it far more difficult to perform a large-scale chemotherapy over several consecutive years in the field.

The main concomitant measures here were categorized into three groups only. As health education for schistosomiasis and its control could be implemented through different paths or mediums such as school-training, word-of-mouth, broadcast, TV, and so on, the community-wide treatment program usually involved such work with the purpose of improving chemotherapy coverage. Chemotherapy in all or infected bovines could reduce the contamination of snail areas with excreted eggs, particularly in endemic areas where bovines are regarded as main reservoirs [55], and snail control could reduce infected snail density. Both can decrease risk of infection for humans. We here observed the higher control effect for the chemotherapy program combined with bovine chemotherapy plus snail control than for the others. However, the difference was not significant. The main explanation could be that the extant to these measures carried out or their quality could not be characterized quantitatively. Indeed, we still face the challenge for treatment of bovines less than six months old as they are not under control, and moreover, the responsibility for bovine chemotherapy in China is taken by agriculture department rather than by health department [56]; we could not find all ‘hot spots’ (i.e. sites with infected snails) based on the current or the renovated snail survey method [57], and snail control targeted at sites contributing most to transmission can be very efficient but, conversely, will be ineffective if any of these sites are missed [58]; the specific contribution of health education within integrated programs for schistosomiasis control awaits assessment [59]. All these may make a sound comparison unavailable between ACWT combined with different measures.

Schistosomiasis japonica is mainly prevalent in lake, marshland and mountainous regions of China [35] and it is well predicted that control effects of ACWT could be possibly in relation to various endemic settings. We here indeed observed a significant influence of the factor in individual variable analyses, although multivariate analysis did not obtain consistent results which is partly due to small numbers of studies available. The evidence for the impact of different endemic regions seemed stronger when subgroup analyses were performed, from which the control effect was significantly higher in mountainous regions than in others. Previous research had suggested that coverage of mass chemotherapy was one of the most directly-related factors to the prevalence in humans [60]. However, in our research we observed no consistent results on this aspect between one and two rounds, mainly due to a considerably high coverage of mass chemotherapy across most studies. We also did not find any significant and consistent association between the effect and targeted community population size or initial prevalence. Regarding any other potential confounding factors, we lack enough references to make further analyses on this aspect.

Although ACWT, alone or combined with other measures, has shown a significant preventive effect against schistosome infections, there is considerable concern that this might result in the development of drug-resistant schistosomes. For example, the low cure rates of 18–38% of *S. mansoni* infections reported in Senegal of Africa have raised worries for emergence of resistance to the drug [61]. However, no evidence of drug resistance on *S. japonicum* has so far been reported [62], [63], which indicated the usefulness and stability of mass chemotherapy in the near term in China or other similar endemic countries.

There are limitations to our study. First, infection intensity is an important aspect of schistosomiasis, and the treatment could make a reduction of over 95% in terms of mean egg counts [64]. However, most studies included in our research reported no data on egg or miracidia counts, and the others reported either arithmetic or geometric means, based on infected individuals or whole population, with no standard deviation. Therefore, the estimation of the control effect in reduction of infection intensity was not possible. Secondly, the stool examination techniques such as Kato-Katz and hatching tests have different sensitivities. At low levels of infection the sensitivity of the Kato-Katz test was especially poor, although a recent report showed the same issue for the hatching test [65]. Different diagnostic methods used could have impact on the estimated size effect. Finally, the information on control measures prior to the research performed was not presented for all included studies and then was not taken into account when this meta-analysis was performed. This might result in an overestimate of the control effect. See Table S1.

There are, however, potential benefits from annual community-wide chemotherapy, which could have long been neglected. This chemotherapy program could eliminate both mixed-sex and single-sex infections of schistosome in humans. At present, the very low infection prevalence of *S. japonicum* in snail populations, for example down to 0.14% [66] in 2011, and a very high proportion (for example up to 95.65%, unpublished data) of single-sex infections of infected snails indicate that in the endemic areas of China, final hosts including humans are more likely to be infected with single-sex schistosome, either males or females. However, the currently used diagnosis methods, parasitological or serological tests [67], are not able to detect such infections, therefore leaving a majority of infections unidentified. As unmated male or female worms are able to live for at least one year within a final host [68], any incoming parasites through infection within the next year, if with an opposing sex, could mate with the previous worms and then complete sexual reproduction, thus resulting in pathology and possible transmission. This scenario could be more common today as the mobile people are on the increasing following social and economical rapid development.

Schistosomiasis control and elimination emphasizes the need for integrated approaches [10], [69], but it is difficult and costly to sustain such programs (see the debate [70], [71]), particularly in low- or middle-income countries. The results from this analysis demonstrated that the control effect of annual community-wide treatment (alone or with other measures) against the parasite in humans was statistically significant. This effect increased with rounds carried out, and was also in relation to categories of endemic regions where it was performed. Although there is a general consensus among both health workers and policy makers that the criteria for annual community-wide treatment should change over time in response to the current infection status [3], [5], the evaluation of such criteria merits further research, as infection prevalence in humans slowly increased once large-scale chemotherapy stopped [72], plus the factor that such chemotherapy has a potential benefit in reducing single-sex infections.

## Supporting Information

Figure S1 **The sensitivity analysis for one round of ACWT against schistosome in humans.** PR, ratio of infection prevalence after one round of ACWT to prevalence before when excluding the study. ACWT refers to annual community-wide treatment.
(TIF)

Figure S2 **The sensitivity analysis for two consecutive rounds of ACWT against schistosome in humans.** PR, ratio of infection prevalence after two rounds of ACWT to prevalence before when excluding the study. ACWT refers to annual community-wide treatment.
(TIF)

Figure S3 **The sensitivity analysis for three consecutive rounds of ACWT against schistosome in humans.** PR, ratio of infection prevalence after three rounds of ACWT to prevalence before when excluding the study. ACWT refers to annual community-wide treatment.
(TIF)

Table S1 **Summary of the characteristics of the included studies in our meta-analyses.**
(XLS)

Checklist S1 **PRISMA 2009 Checklist.**
(DOC)
