# Supplementary figures and images for: Control Efficacy of Annual Community-Wide Treatment against Schistosoma japonicum in China: A Meta-Analysis
Source: PLoS One. 2013 Nov 4;8(11):e78509. doi: 10.1371/journal.pone.0078509 (PMC3817216; doi:10.1371/journal.pone.0078509)

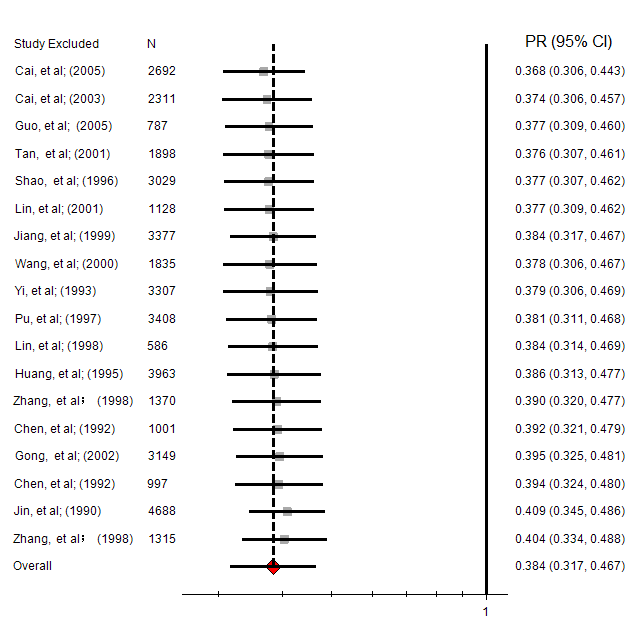

Supplement: Figure S1 — The sensitivity analysis for one round of ACWT against schistosome in humans. PR, ratio of infection prevalence after one round of ACWT to prevalence before when excluding the study. ACWT refers to annual community-wide treatment. (TIF) [file pone.0078509.s001.tif]

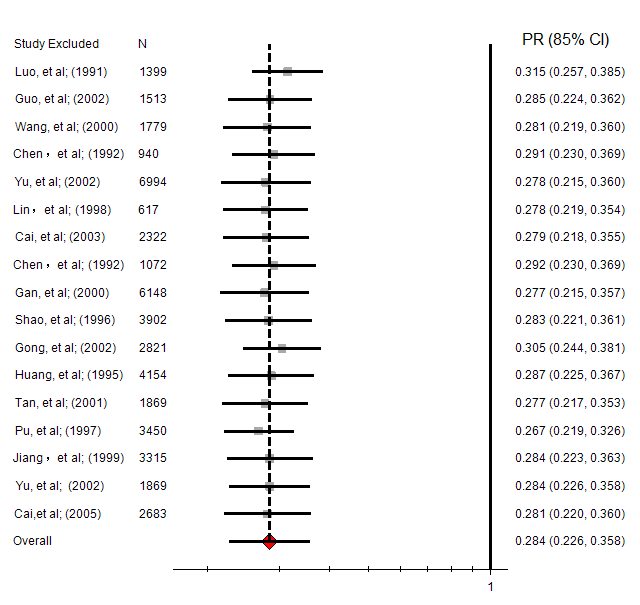

Supplement: Figure S2 — The sensitivity analysis for two consecutive rounds of ACWT against schistosome in humans. PR, ratio of infection prevalence after two rounds of ACWT to prevalence before when excluding the study. ACWT refers to annual community-wide treatment. (TIF) [file pone.0078509.s002.tif]

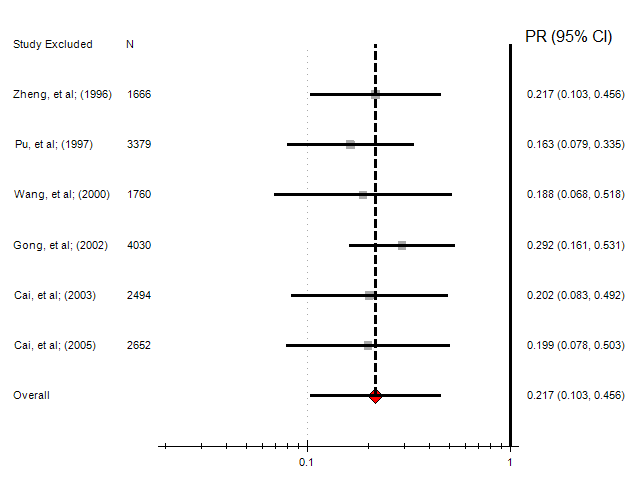

Supplement: Figure S3 — The sensitivity analysis for three consecutive rounds of ACWT against schistosome in humans. PR, ratio of infection prevalence after three rounds of ACWT to prevalence before when excluding the study. ACWT refers to annual community-wide treatment. (TIF) [file pone.0078509.s003.tif]
